# Totally Spin-Polarized Currents in an Interferometer with Spin–Orbit Coupling and the Absence of Magnetic Field Effects

**DOI:** 10.3390/nano12224082

**Published:** 2022-11-20

**Authors:** Victor Lopes, Guillermo Chiappe, Laercio C. Ribeiro, Enrique V. Anda

**Affiliations:** 1 Departamento de Física Aplicada, Universidad de Alicante, San Vicente del Raspeig, 03690 Alicante, Spain; 2 Centro Federal de Educação Tecnológica Celso Suckow da Fonseca CEFET/RJ, Campus Nova Iguaçu, Nova Iguaçu, Rio de Janeiro 26041-271, Brazil; 3 Departamento de Física, Pontifícia Universidade Católica do Rio de Janeiro (PUC-Rio), Rio de Janeiro 22451-900, Brazil

**Keywords:** spin–orbit coupling, spintronics, interferometer, functional nano-heterostructures, one-dimensional nanostructures, semiconductor nanowires, spin-polarized current

## Abstract

The paper studies the electronic current in a one-dimensional lead under the effect of spin–orbit coupling and its injection into a metallic conductor through two contacts, forming a closed loop. When an external potential is applied, the time reversal symmetry is broken and the wave vector *k* of the circulating electrons that contribute to the current is spin-dependent. As the wave function phase depends upon the vector *k*, the closed path in the circuit produces spin-dependent current interference. This creates a physical scenario in which a spin-polarized current emerges, even in the absence of external magnetic fields or magnetic materials. It is possible to find points in the system’s parameter space and, depending upon its geometry, the value of the Fermi energy and the spin–orbit intensities, for which the electronic states participating in the current have only one spin, creating a high and totally spin-polarized conductance. For a potential of a few tens of meV, it is possible to obtain a spin-polarized current of the order of μA. The properties of the obtained electronic current qualify the proposed device as a potentially important tool for spintronics applications.

## 1. Introduction

The possibility of controlling the degree of freedom of electronic spin has been very important for the development of spintronics [1,2,3,4,5] and is very promising for the elaboration of qubits devices in the field of quantum computing [6,7,8]. In this context, several systems have been proposed as sources of spin-polarized current, most of which use the coupling of electron motion and spin, through spin–orbit coupling (SOC) [9]. This is the case of the Datta and Das transistor [10] based on the spin precession of the electronic current produced by this interaction in a narrow-gap semiconductor located between two magnetized contacts and devices [11,12,13,14] based on persistent spin helix states [15]. However, the spin splitting that could facilitate the creation of polarized currents cannot, in principle, be produced by the unique action of SOC, because the system possess states with opposite spins and the same energy due to Kramer’s degeneracy [16], a result of time reversal symmetry [9]. Magnetic fields, magnetic materials, and the injection of spin-polarized currents [17,18,19,20,21,22,23,24,25,26,27] or time-dependent Hamiltonians [28] have been used to break this symmetry and generate or manipulate spin-polarized currents. To obtain this result in non-magnetic materials, or without applying an external magnetic field, several systems have been proposed, such as point contact [29,30], graphene nanostructures [31], planar systems with SOC and corrugated graphene nanoribbons [32,33,34], or even creating spin-dependent chemical potentials by microwave irradiation [35]. In reference [29], the authors reported a plateau in the conductance of quantum point contact (QPC) devices, which was suggested to be intrinsically related to the spontaneous spin polarization induced by lateral SOC for sufficiently high asymmetry of the lateral confinement. The asymmetry was induced by the difference in the gate voltages applied through contacts that were transverse to the electronic current direction. The conductance was measured as a function of a “sweeping voltage”, simultaneously applied to all gates of the device. In another paper [36], some of the authors theoretically handled the problem of spin polarization associated with lateral spin orbit coupling by controlling the asymmetry of the lateral confinement potential. The authors reported a spin-polarized conductance derived from such asymmetry but did not obtain a totally spin-polarized current. The maximum polarization obtained was 60%. This result was attributed to the single-particle model adopted, which does not incorporate the electron–electron interaction, suggested as one of the essential ingredients to obtain totally spin-polarized conductance [37]. Mikio Eto et. al. [38] also obtained a spin polarization of more than 50% in a QPC system with Rashba SOC in the absence of a magnetic field or magnetic materials, for experimentally accessible SOC intensities.

The authors of a recent paper [39] reported that some chiral molecules, such as DNA, when placed between two metal contacts, can operate as a spin filter device, placing emphasis on the orbital texture of the band structure and its influence in the polarization of the quantum orbitals, which depends on the molecule chirality. This induces spin polarization, due the spin–orbit effect in the metal contacts, in a process called chiral-induced spin selection (CISS). In reference [40], the authors considered a system with SOC composed of two QPC, one of them working as a collector—a spin analyzer—of the electrons emitted from the other. In this device, the electrons emitted from the first QPC are focused onto the collector through an external magnetic field perpendicular to the two-dimensional electron gas. The charge accumulation in the collector produces voltage differences and peaks through which, for the system with Rashba SOC, the spin projection of the electrons leaving the emitter can be detected. Specifically, the spin polarization of the electronic current is proportional to the height of two subsequent voltage peaks. The authors obtained a spin polarization of 70%.

In addition, there are devices based on the Aharonov–Bohm effect [41], where a magnetic flux through a ring breaks the time inversion symmetry. Many authors have argued about this type of system, proposing spin-interference devices with the Aharonov–Bohm effect and SOC [17,42,43,44,45,46,47,48,49], rings with a magnetization that gradually varies [50], or rings irradiated by an electromagnetic field [51], creating an interference pattern reflected in the current that circulates along the system. It is important to note that the correct form of Hamiltonian to study this type of system, composed by the mesoscopic ring and the SOC, was not always used. For a detailed discussion of this problem, see refs. [43,47].

Although there have been a large number of different proposals to produce spin-polarized current devices, the polarization is not always easily manipulated or modified.

In this paper, we study the conduction properties of a device consisting of a one-dimensional lead with spin–orbit coupling (1DLSOC), connected by two contacts, located at two different sites of the lead, to a metallic conductor, creating a circuit with a closed loop. The SOC and the broken time reversal symmetry produced by the applied voltage give rise to a spin-dependent interference among the wave functions that describes the electrons circulating along the two branches of the system. It is possible to show that the conductance can be high and even completely spin-polarized, while the electronic current injected into the metallic lead could reach μA for a potential of the order of meV. The idea of creating a spin-polarized source using the broken symmetry produced by an external bias has also been recently studied in a colinear antiferromagnetic device. The external bias breaks the spin sublattice’s symmetry, generating a spin-polarized current, with a polarization depending on the magnitude of the external bias voltage applied. The polarization obtained was nearly 80% [52]. The system we propose can operate with high and totally spin-polarized conductance, with relatively small SOC intensities. Moreover, the device does not require the use of magnetic fields or magnetic materials, which facilitates the injection of the polarized current into a metallic lead.

## 2. Interferometer Design and Model Description

The system is described by a 1D tight-binding Hamiltonian that, even under the effect of SOC, possesses SU(2) symmetry. This property permits a quantization axis to be defined in an $\hat{r}$ direction, determined by the intensities of the Rashba and Dresselhaus SOC, along which the spin is a good quantum number. This allows a SOC pseudo magnetic field to be defined, depending upon the linear momentum *k* of the electron and pointing in the $\hat{r}$ direction. The pseudo magnetic field points in the opposite direction when *k* changes to −k, conserving time reversal symmetry [53]. However, when an external voltage is applied, this symmetry is broken because the degenerate states with momentum *k* and −k and opposite spin directions are differently populated. The occupied *k* values of the electrons that contribute to the current introduce a spin-dependent wave function phase, which produces different interference patterns in the closed loop of the circuit, creating a spin-polarized current.

**Figure 1:**
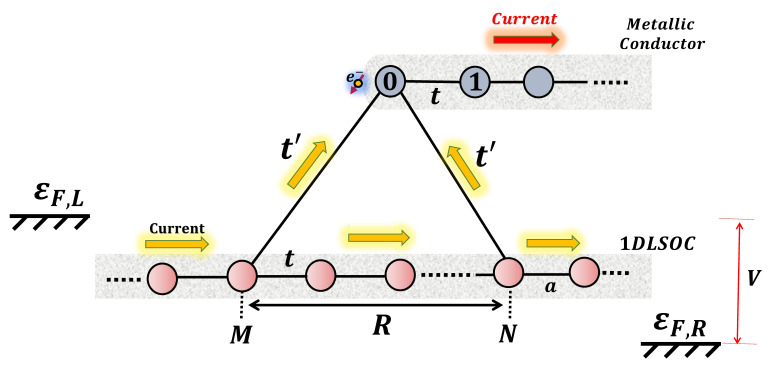
Diagram of the interferometer composed of a one-dimensional lead on the SOC side coupled through two contacts to a metallic conductor.

The system proposed is shown in Figure 1. It possesses a side-connected metallic lead, represented by a half-linear chain into which the spin-polarized current is injected. This metallic conductor is connected to a 1DLSOC by two contacts separated by a distance *R*, through which the electrons can circulate. The wave function phase interference of the electrons contributing to the current, depending upon the parameters, can give rise to a total destructive interference for just one spin state, resulting in a fully spin-polarized current for the opposite spin.

The 1DLSOC could be a nanowire adsorbed onto a metallic surface with a strong or giant Rashba SOC [54,55,56,57,58,59,60,61,62,63,64,65,66] or a semiconductor nanowire, as in the systems studied in refs. [67,68]. An external electrical field, along the perpendicular direction of the 1DLSOC, enables the enhancement of the Rashba SOC intensity, allowing the phase difference to be controlled between electrons with different spins. Moreover, this can be obtained by manipulating the Fermi energies of the conductors through the application of a gate potential on the metallic substrate, changing its charge content. The external voltage, *V*, corresponds to the difference between the left and right sides of the Fermi device energies given by $\epsilon_{F,L}=\epsilon_{F}+V\;/\;2$ and $\epsilon_{F,R}=\epsilon_{F}-V\;/\;2$, respectively. The energy $\epsilon_{F}$ defines the Fermi level in thermodynamic equilibrium, when V=0.

The Hamiltonian that describes the system is given by

(1) $$H=\sum_{k,\sigma_{r}}\epsilon_{k\sigma_{r}}c_{k\sigma_{r}}^{†}c_{k\sigma_{r}}+\sum_{<i,j>,\sigma_{r}}t_{ij}f_{i\sigma_{r}}^{†}f_{j\sigma_{r}}+\sum_{k,\sigma_{r}}\left(t_{k}^{\prime }f_{0\sigma_{r}}^{†}c_{k\sigma_{r}}+h.c.\right),$$

where the first term, in the reciprocal space representation, corresponds to the 1DLSOC, with a dispersion relation [53]

(2) $$\epsilon_{k\sigma_{r}}=-2z\cos\left(ka-\sigma_{r}\psi \right).$$

The renormalized nearest-neighbor hopping of the 1DLSOC is $z=\sqrt{t^{2}+{\left|\gamma \right|}^{2}}$, where γ=β+iα, $i=\sqrt{-1}$, α and β being the Rashba and Dresselhaus SOC intensities, respectively. The parameter *t* is the nearest-neighbor hopping in the 1DLSOC and in the metallic conductor, which, for simplicity, has been taken to be equal, and *a* is the lattice parameter. The spin, represented by the quantum number $\sigma_{r}$, is quantized along an axis pointing in the direction $\hat{r}\left(\theta ,\phi \right)=\hat{r}\left(\pi \;/\;2,\phi \right)$, where θ is the polar angle and $\phi =\tan^{-1}\left(\alpha \;/\;\beta \right)$ [53]. The operator $c_{k\sigma_{r}}^{†}\left(c_{k\sigma_{r}}\right)$ creates (annihilates) an electron with momentum *k* and spin $\sigma_{r}$ in the 1DLSOC. The second term describes the side-attached metallic conductor and the third corresponds to the connection between both subsystems. The operator $f_{i\sigma_{r}}^{†}$$\left(f_{i\sigma_{r}}\right)$ creates (annihilates) an electron at site *i* of the metallic conductor. The matrix elements connecting the two subsystems, when the Hamiltonian describing the 1DLSOC is represented in the reciprocal space, carry a *k*-dependent phase, which is given by $t_{k}^{\prime }=t^{\prime }\left(1+\exp\left(ikR\right)\right)$, where *R* is the distance between the sites *N* and *M* of the SOC chain where the connections are established, as shown in Figure 1, and $t^{\prime }$ is supposed to be site-independent.

It is important to emphasize that $\hat{r}$ determines the direction of the spin polarization of the current circulating through the device. It can be manipulated by modifying the intensity parameter α of the Rashba SOC. The phase $\psi =\tan^{-1}\left(\left|\gamma \right|\;/\;t\right)$, which appears in Equation (2), is responsible for the spin-dependent occupation of the *k* states when the applied voltage *V* drives the system out of thermodynamic equilibrium regime. Moreover, the phase difference in the dispersion relation between spin up and down, ψ, gives rise to the spin and momentum *k*-dependent interference among the charges contributing to the current.

**Figure 2:**
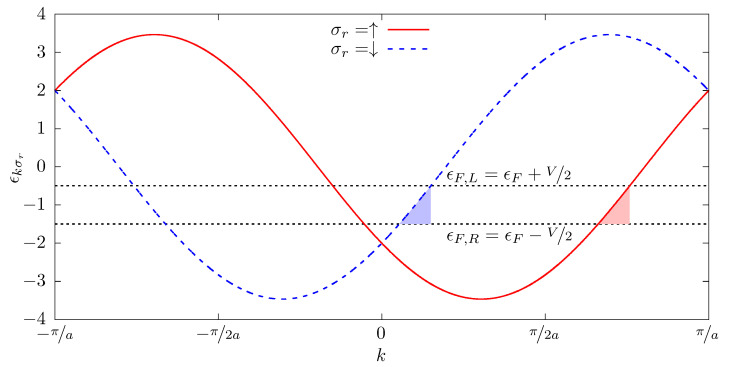
Dispersion relation $\epsilon_{k\sigma_{r}}$ as a function of *k* and spin $\sigma_{r}$. The SOC intensities are taken to be α=β=1t. The colored shadows illustrate, for each spin and $\epsilon_{F,R}<\epsilon <\epsilon_{F,L}$, the electronic states that participate in the current.

In order to illustrate the influence of the SOC, we show in Figure 2 the dispersion relation $\epsilon_{k\sigma_{r}}$ as a function of *k* and spin $\sigma_{r}$, where the SOC intensities are taken to be α=β=1t. It is shown that for a value of $\epsilon_{k\sigma_{r}}$ between $\epsilon_{F,R}$ and $\epsilon_{F,L}$, electrons with different spins contribute to the current when $\partial \epsilon_{k\sigma_{r}}\;/\;\partial k>0$, with different *k* values. In the figure, *k* regions are represented by colored shadows.

## 3. Current and Conductance Calculation

To calculate the conductance and the current injected into the metallic lead, we use the Keldysh formalism [69,70], appropriated to study systems out of thermodynamic equilibrium. In order to explore the advantages of dealing with the exact solution of the SOC linear chain, we consider the two couplings connecting, through $t^{\prime }$, the metallic semi-chain to the *k* states that represent the 1DLSOC, as assumed in the Hamiltonian, Equation (1). The occupation of these states depends upon the Fermi energies $\epsilon_{F,R}$ and $\epsilon_{F,L}$. For energies $\epsilon <\epsilon_{F,R}$, all of the *k* states are occupied, while for the interval $\epsilon_{F,R}<\epsilon <\epsilon_{F,L}$ the occupied *k* states, due to the direction of the current, are those that fulfill the spin-dependent condition, $\partial \epsilon_{k\sigma_{r}}\;/\;\partial k>0$. With this in mind, we calculate $J^{\sigma_{r}}\left(V\right)$, the spin-dependent electronic current between sites 0 and 1, that corresponds to the current injected into the metallic conductor. This is given by

(3) $$J^{\sigma_{r}}\left(V\right)=\frac{e}{h}t\int_{\epsilon_{F}-V\;/\;2}^{\epsilon_{F}+V\;/\;2}d\epsilon \left(\overset{-+}{G_{01}^{\sigma_{r}}\left(\epsilon \right)}-\overset{-+}{G_{10}^{\sigma_{r}}\left(\epsilon \right)}\right),$$

where $\overset{-+}{G_{ij}^{\sigma_{r}}}\left(\epsilon \right)$ are the out of equilibrium Green functions [70] that depend upon the Fermi levels $\epsilon_{F,R}=\epsilon_{F}-V/2$ and $\epsilon_{F,L}=\epsilon_{F}+V/2$ and on the spin $\sigma_{r}$. The $\overset{-+}{G_{ij}^{\sigma_{r}}}\left(\epsilon \right)$ are obtained through a perturbation calculation that considers the unperturbed system, in thermodynamic equilibrium, corresponding to the structure when the connections, of site 0 with *k* states of 1DLSOC and with site 1 of the metallic contact, are eliminated. This satisfies the relationship

(4) $$\overset{-+}{G}\left(\epsilon \right)=\left(1+G^{\left(r\right)}\left(\epsilon \right)\sum \right)\overset{-+}{g}\left(\epsilon \right)\left(1+\sum G^{\left(a\right)}\left(\epsilon \right)\right),$$

where the non-equilibrium matrix $\overset{-+}{G}\left(\epsilon \right)$ is written as a function of the retarded and advanced Green functions, $G^{\left(r,a\right)}\left(\epsilon \right)$, superscript r and a, respectively, of the complete structure and $\overset{-+}{g}\left(\epsilon \right)$ that corresponds to the unperturbed system. The matrix ∑ is the self-energy resulting from the connections of site 0 with the rest of the system.

The current is calculated from Equations (3) and (4). It is obtained from the expression

(5) $$J^{\sigma_{r}}\left(V\right)=\frac{e}{h}2t^{2}t^{\prime 2}\int_{\epsilon_{F}-V\;/\;2}^{\epsilon_{F}+V\;/\;2}d\epsilon {\left|G_{00}\left(\epsilon \right)\right|}^{2}\sum_{k}\left(1+\cos(kR)\right)\overset{-+}{g_{k}^{\sigma_{r}}}\left(\epsilon \right)\overset{+-}{g_{1}}\left(\epsilon \right),$$

where $\overset{-+}{g_{1}}\left(\epsilon \right)=2\pi \rho_{1}\left(\epsilon \right)f\left(\epsilon_{F,R}\right)$, $\rho_{1}\left(\epsilon \right)=\sqrt{4t^{2}-\epsilon^{2}}\;/\;2\pi t^{2}$ and $f(\epsilon_{F,R})$ is the Fermi distribution with a Fermi energy $\epsilon_{F,R}$. The undressed equilibrium Green function $\overset{-+}{g_{k}^{\sigma_{r}}}\left(\epsilon \right)=2\pi \rho_{k}^{\sigma_{r}}f\left(\epsilon_{F,k}\right)$ depends upon the linear momentum such that the sum of *k* is calculated considering that when $\partial \epsilon_{k\sigma_{r}}\;/\;\partial k>0$, $\epsilon_{F,k}=\epsilon_{F,L}$, while for $\partial \epsilon_{k\sigma_{r}}\;/\;\partial k<0$, $\epsilon_{F,k}=\epsilon_{F,R}$ and $\rho_{k}^{\sigma_{r}}\left(\epsilon \right)=\delta \left(\epsilon -\epsilon_{k\sigma_{r}}\right)$. The function δ(ϵ) is Dirac’s delta function.

The equilibrium Green function at site 0 can be written as

(6) $$G_{00}\left(\epsilon \right)=\frac{1}{\epsilon -t^{2}g_{1}\left(\epsilon \right)-2t^{\prime 2}\sum_{k}\left(1+\cos(kR)\right)g_{k}^{\sigma_{r}}\left(\epsilon \right)},$$

where $g_{1}\left(\epsilon \right)=\left(\epsilon \pm \sqrt{\epsilon^{2}-4t^{2}}\right)\;/\;2t^{2}$ and $g_{k}^{\sigma_{r}}\left(\epsilon \right)=1\;/\;\left(\epsilon -\epsilon_{k\sigma_{r}}-i\eta \right)$ are the undressed equilibrium Green functions at the first site of the semi-chain and at the *k* momentum state of the 1DLSOC, respectively. The summation that appears in the denominator of Equation (6) is over all *k*’s belonging to the first Brillouin zone. This is why the Green function $G_{00}\left(\epsilon \right)$ is spin-independent. However, the summation in *k* of Equation (5) is restricted to the momentum of the electrons participating in the current, satisfying $\partial \epsilon_{k\sigma_{r}}\;/\;\partial k>0$. This condition originates a spin-dependent current result from Equation (5).

The conductance is obtained from the equations

(7) $$J^{\sigma_{r}}\left(V\right)=\frac{1}{e}\int_{\epsilon_{F}-V\;/\;2}^{\epsilon_{F}+V\;/\;2}d\epsilon G^{\sigma_{r}}\left(\epsilon \right)$$

and

(8) $$G^{\sigma_{r}}\left(\epsilon \right)=\frac{e^{2}}{h}8t^{2}t^{\prime 2}\pi^{2}{\left|G_{00}\left(\epsilon \right)\right|}^{2}z\frac{1+\cos\left[k_{+}^{\sigma_{r}}\left(\epsilon \right)R\right]}{\sqrt{4z^{2}-\epsilon^{2}}}\rho_{1}\left(\epsilon \right),$$

where

(9) $$k_{+}^{\sigma_{r}}\left(\epsilon \right)=\frac{1}{a}\left[\cos^{-1}\left(-\frac{\epsilon }{2z}\right)+\sigma_{r}\psi \right]$$

is the momentum wave vector, restricted by the condition $\partial \epsilon_{k\sigma_{r}}\;/\;\partial k>0$.

The phase of the 1DLSOC Bloch wave function $\phi \left(x\right)=\exp\left(ikx\right)u_{k}\left(x\right)$ that defines the interference pattern in the closed loop of the circuit depends upon the *k* vector. This is reflected by the $\cos(k_{+}^{\sigma_{r}}\left(\epsilon \right)R)$ dependence of the conductance as it appears in Equation (8). Analyzing this equation, it is possible to conclude that, for a given value of *R*, the conductance with spin $\sigma_{r}$ is zero when $k_{+}^{\sigma_{r}}\left(\epsilon \right)R=n\pi$, where *n* is an odd integer. Hence, as the value of $k_{+}^{\sigma_{r}}\left(\epsilon \right)$ that contributes to the current depends upon the Fermi energy $\epsilon_{F}$ and on the spin–orbit interaction phase φ, the conductance oscillates, modifying these parameters. It is clear that the frequency of the oscillation increases with *R*. The polarization of the current is mainly a result of the dependence of $k_{+}^{\sigma_{r}}\left(\epsilon \right)$ on $\sigma_{r}$, as shown in Equations (8) and (9). Finally, for the conductance to be completely spin-polarized, the Fermi level $\epsilon_{F}$ should satisfy

(10) $$\epsilon_{F}=-2z\cos\left(\frac{n\pi a}{R}-\sigma_{r}\psi \right).$$

In order to investigate the conditions that optimize the device performance, we calculate the differences between the conductances for opposite spins, finding that

(11) $$\begin{array}{cc} |G^{\sigma_{r}}\left(\epsilon \right)-G^{\bar{\sigma_{r}}}\left(\epsilon \right)|= & \frac{e^{2}}{h}8t^{2}t^{\prime 2}\pi^{2}{\left|G_{00}\left(\epsilon \right)\right|}^{2}z\frac{\rho_{1}\left(\epsilon \right)}{\sqrt{4z^{2}-\epsilon^{2}}}\times \\ \; & 2\sin\left[\frac{R}{2}\left(k_{+}^{\sigma_{r}}+k_{+}^{\bar{\sigma_{r}}}\right)\right]\sin\left[\frac{R}{2}\left(k_{+}^{\sigma_{r}}-k_{+}^{\bar{\sigma_{r}}}\right)\right]. \end{array}$$

Analyzing this equation, we can see that the difference between the two opposite spin conductances is at its maximum when the sine arguments are multiples of π/2, i.e., $(k_{+}^{\sigma_{r}}+k_{+}^{\bar{\sigma_{r}}})R/2=l\pi /2$ and $(k_{+}^{\sigma_{r}}-k_{+}^{\bar{\sigma_{r}}})R/2=m\pi /2$, where *l* and *m* are odd integers. According to the first condition, the Fermi level $\epsilon_{F}$ satisfies the relationship

(12) $$\epsilon_{F}=-2z\cos\left(\frac{l\pi a}{2R}\right),$$

while the second determines that the Rashba SOC intensity α is given by

(13) $$\alpha ={\left[t^{2}\tan^{2}\left(\sigma_{r}\frac{m\pi a}{R}\right)-\beta^{2}\right]}^{1\;/\;2}.$$

**Figure 3:**
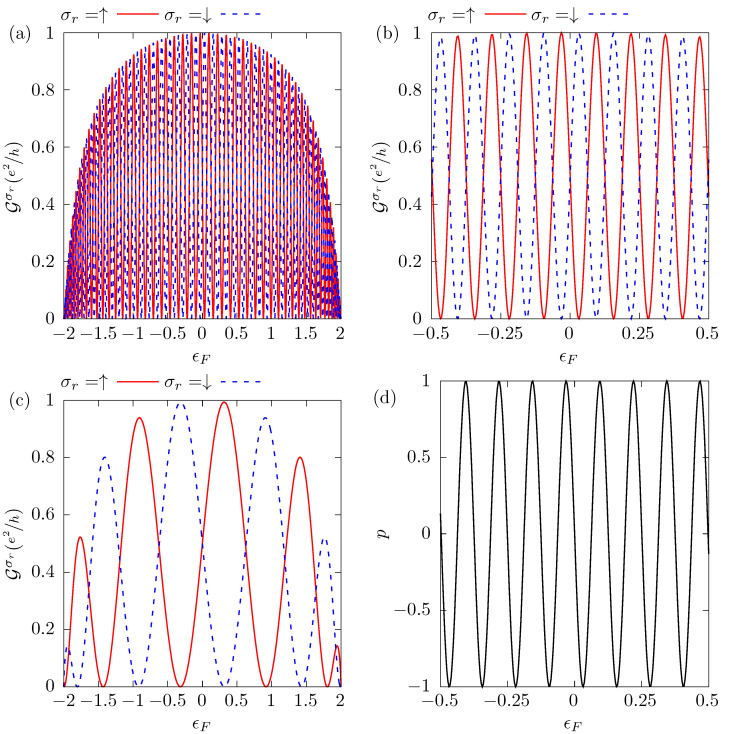
The conductance $G^{\sigma_{r}}$ for each spin $\sigma_{r}$, panels (**a**–**c**), and the spin polarization of the current *p*, panel (**d**), as a function of the Fermi energy $\epsilon_{F}$. Panels (**a**,**b**,**d**) correspond to a system with R=100a, $\alpha =1.212\times 10^{-2}t$ and $\beta =10^{-2}t$, while in panel (**c**) the parameters are R=10a, α=0.158t, and $\beta =10^{-2}t$. For both configurations, $t^{\prime }=t$.

It is important to emphasize that Equations (10) and (12) are compatible if Equation (13) is satisfied, indicating that the maximum polarized current is obtained when it is completely spin-polarized. The conditions expressed in Equations (12) and (13) determine the Fermi level $\epsilon_{F}$ required to obtain a complete spin polarization of the current. In other words, this double condition maximizes the difference between the conductances, thus meaning that for one spin the conductance is zero and for the other it adopts the maximum possible value, a half quantum of conductance, $e^{2}\;/\;h$. This is the case shown in Figure 3, where the conductance $G^{\sigma_{r}}\left(\epsilon_{F}\right)$ oscillates, as a function of the Fermi level $\epsilon_{F}$, between zero and $e^{2}\;/\;h$. We also observe in Figure 3a,c that the conductance peaks, around $\epsilon_{F}=0$, are higher than those at the edges of the band due to the dependence of the conductance on the DOS of the metallic semi-chain (see Equation (8)). We present in Figure 3b a magnification, around the origin, of the same results as in Figure 3a, where the totally polarized conductance maximum value is half a quantum of conductance. In this configuration, the parameters of the system are taken to be R=100a, $t^{\prime }=t$ and we assume the Dresselhaus spin–orbit interaction $\beta =10^{-2}t$. According to Equation (13), a completely spin-polarized current with conductance equal to $e^{2}\;/\;h$ is achieved when the Rashba SOC is $\alpha ≅1.212\times 10^{-2}t$, which can be obtained by controlling the external electric field responsible for the Rashba SOC, operating as a device tuner. Panel (**c**) shows the conductance for a small value of the distance between contacts, R=10a, and $\beta =10^{-2}t$. In this configuration, the value of α that satisfies the full spin polarization conditions is α=0.158t. The Rashba SOC α manipulation ensures that when the conductance for one spin is zero, the conductance for the other could be very close to the maximum possible value, such that the device operates with a large and fully polarized current.

The amount of oscillation presented in the conductance results from the product $k_{+}^{\sigma_{r}}\left(\epsilon \right)R$. The number of oscillations increases with the distance between the contacts, *R*, as we can see in Figure 3a for R=100a and in Figure 3c for R=10a, where it is also observed that the peaks reach their maximum values in the region close to $\epsilon_{F}=0$. The spin polarization *p* is shown in Figure 3d. This quantity, defined as

(14) $$p\left(\epsilon \right)=\frac{G^{↑}\left(\epsilon \right)-G^{↓}\left(\epsilon \right)}{G^{↑}\left(\epsilon \right)+G^{↓}\left(\epsilon \right)},$$

reflects the oscillatory behavior of conductance, assuming the values +1 and −1 when the current is totally polarized in the spin-up and spin-down directions, respectively.

**Figure 4:**
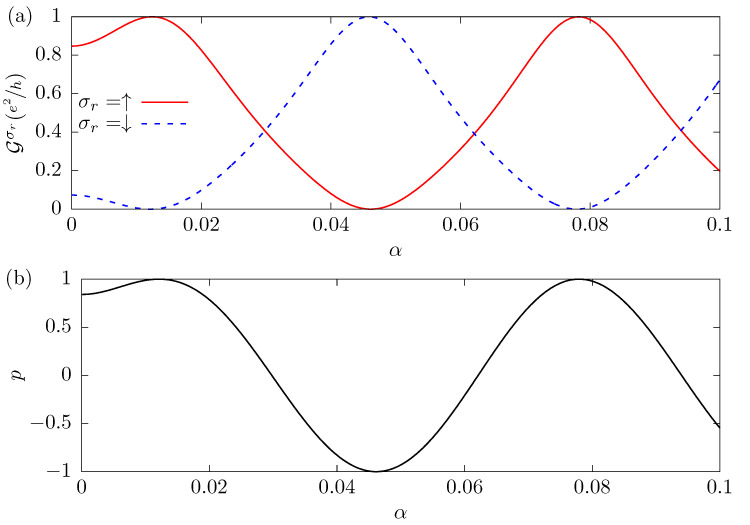
The conductance $G^{\sigma_{r}}$, panel (**a**), and the spin polarization of the current *p*, panel (**b**), as a function of the Rashba SOC intensity α. The distance between contacts is R=100a and the Dresselhaus SOC parameter is $\beta =10^{-2}t$. The Fermi level is given by Equation (12), $\epsilon_{F}=9.422\times 10^{-2}t$, and when α satisfies Equation (13), $G^{\sigma_{r}}$ is completely spin-polarized.

Figure 4 shows the conductance $G^{\sigma_{r}}$, panel (**a**), and the spin polarization *p*, panel (**b**), as a function of the Rashba SOC intensity α, for the parameters R=100a, $t^{\prime }=t$, and $\beta =10^{-2}t$. The Fermi level is assumed to be $\epsilon_{F}=9.422\times 10^{-2}t$ and corresponds to the first positive value where the spin-down conductance is zero, as shown in Figure 3b. Analyzing the results, we see that by increasing the Rashba SOC intensity and keeping the other parameters fixed, $G^{↓}$ and $G^{↑}$ change their value until $G^{↑}$ becomes close to $e^{2}\;/\;h$ and $G^{↓}=0$, when $\alpha =1.212\times 10^{-2}t$. This α value satisfies the condition imposed by Equation (13), reproducing the maximum of $G^{↑}$ and the minimum of $G^{↓}$ as shown in Figure 3b. We notice that by increasing α, the $G^{\sigma_{r}}$ oscillates and other points of maximum polarization appear with conductance near $e^{2}\;/\;h$, which corresponds to other solutions of Equation (13). In panel (**b**), when α satisfies Equation (13), we observe that *p* alternatively takes the values of +1 or −1. This figure illustrates that, by changing the Rashba SOC intensity, it is also possible to tune the system so that for one spin the conductance is zero while for the other the results are very close to the maximum possible value.

**Figure 5:**
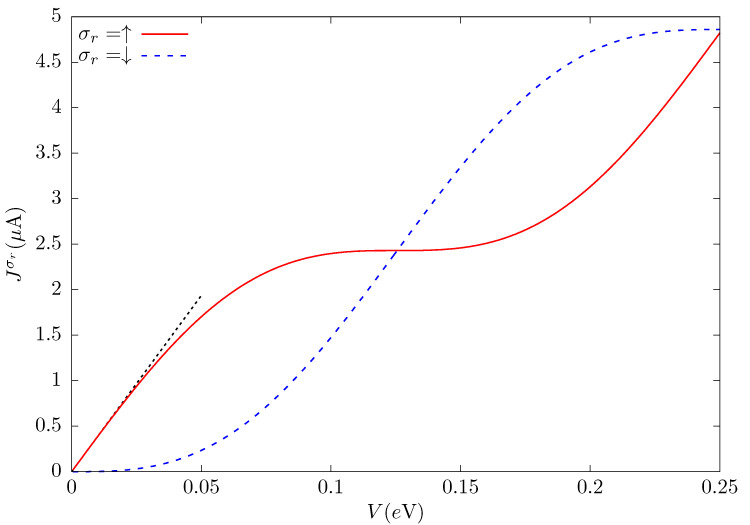
Electronic current $J^{\sigma_{r}}$ injected into the metallic conductor as a function of the applied potential *V*. As in Figure 4, the distance between contacts is R=100a and the Rashba and Dresselhaus SOC parameters are $\alpha =1.212\times 10^{-2}t$ and $\beta =10^{-2}t$, respectively. The Fermi level is taken to be $\epsilon_{F}=9.422\times 10^{-2}t$, value for which the spin-down conductance is zero, and t=1eV. The black dotted line corresponds to the slope e/h.

The electronic current injected into the metallic conductor $J^{\sigma_{r}}\left(V\right)$ is obtained from the conductance $G^{\sigma_{r}}$ by Equation (7). Its behavior, as a function of the applied potential *V*, is shown in Figure 5. The parameters that define the system are taken to be the same as those of Figure 4, for the SOC intensities discussed above, for which the conductance is completely spin-up. As in Figure 4, the Fermi level $\epsilon_{F}$ is fixed at the positive value where the first spin-down conductance minimum occurs, according to Equation (12), $\epsilon_{F}=9.422\times 10^{-2}t$.

As the Fermi level is located at a spin-down conductance minimum, the spin-down current $J^{↓}$ is practically zero until the applied potential increases, V>0.03t, incorporating the integral values of the spin-down conductance that are not zero (Equation (7)). On the other hand, as the spin-up conductance is at a maximum value, the current has an almost linear behavior for small *V*. Increasing the potential *V*, the oscillatory behavior of the conductance begins to influence the current. The spin-down current increases, while the spin-up current reaches a plateau at the center of the figure. Further increasing *V*, the behavior of the spin currents is interchanged until a second plateau appears, close to V=0.25t, for the spin-down conductance.

## 4. Conclusions

We show that a device composed of a one-dimensional lead, under the effect of spin–orbit coupling, connected to a metallic conductor through two contacts that create a closed loop, can operate as a very efficient source of totally spin-polarized current, without requiring the presence of an external magnetic field or magnetic materials. The device permits control of the spin-polarized current by manipulating the intensity of the Rashba spin–orbit coupling and of the Fermi level by the application of an external electric field. In fact, we can tune the degree of polarization of the current in any of the spin directions and obtain a totally spin-polarized conductance very near its maximum value, $e^{2}\;/\;h$, as shown in Figure 3 and Figure 4.

These properties are the result of the pseudo-spin SU(2) symmetry, which permits the definition of a direction of quantization along which the spin is a good quantum number, and of the broken time reversal symmetry produced by the applied potential responsible for the electrical current. An essential ingredient of the proposed device is that its closed loop circuit creates an interference pattern for the circulating electron that determines the total current through the system. The presence of the spin–orbit coupling introduces a spin-dependent phase for the electronic wave function. As a consequence, the interference introduced by the two alternative paths, along which the electronic current circulates, depends upon the spin. This is why, under the effect of an external potential and through an adequate manipulation of the parameters, the device is capable of sustaining high and totally spin-polarized currents. It is important to note that the spin polarization studied corresponds to the current injected into the metallic conductor, which is not directly under the effect of the spin–orbit coupling.

In real materials, disorder is always present. However, there have been several experimental studies in one-dimensional systems with spin–orbit coupling [67,68], which demonstrate that well-defined values of the wave vectors *k* are preserved and that the localization length of the wave function, due to disorder, is much larger than the distances involved in the proper operation of these one-dimensional devices. These devices show high values for conductance, typically corresponding to one-dimensional perfect wires and the existence of a “spin–orbit gap”. This gap is a result of the application of an external magnetic field along the direction of the one-dimensional sample, and is theoretically analyzed by assuming the momentum *k* to be a good quantum number. This shows that in the system we are studying, disorder can be experimentally controlled in order to preserve the transport properties that are described in the manuscript.

Finally, we mention that very interesting transport properties could emerge in more complex systems. This could be the case when electron–electron components with a high degree of interaction, or different geometries in the contact’s design, are incorporated, which could improve the control of the spin-polarized current. Further research along these lines is currently being developed.

## Data Availability

Not applicable.

## Abbreviations

The following abbreviations are used in this manuscript:

SOC	Spin–Orbit Coupling
QPC	Quantum Point Contact
CISS	Chiral-Induced Spin Selective
1DLSOC	One-Dimensional Lead with Spin–Orbit Coupling
